# Did the use of open invitations in place of timed appointment invitations reduce the uptake of breast screening in the London region during the COVID-19 recovery?

**DOI:** 10.1177/09691413221127583

**Published:** 2022-10-11

**Authors:** Sue M Hudson, Kathie Binysh, Stephen W Duffy

**Affiliations:** 1 Peel & Schriek Consulting Limited, London, UK; 2 Head of Screening, 53249 NHS England & Improvement (London), London, UK; 3 Centre for Prevention, Detection and Diagnosis, Wolfson Institute of Population Health, London, UK

**Keywords:** Breast screening, uptake, timed appointments

## Abstract

**Objective:**

The Covid-19 pandemic created a backlog of women awaiting an invitation for breast screening in the UK. To recover in a timely fashion, the National Health Service programme opted to issue open invitations (OI) to women rather than the standard pre-booked timed appointments (TA). Historically, OIs have been shown to result in lower uptake. The aim of this study was to make use of a natural experiment to compare uptake in groups sent an OI with those sent a TA during a period when both invitation methods were in use.

**Methods:**

Women invited for routine screening at one of the six London breast screening services from September 2020 to March 2021 were included and grouped according to the type of invitation they had received (TA or OI). The outcome was attendance within 6 months of opening the screening episode. Data were analysed by logistic regression.

**Results:**

During the period of the study, 78,192 (32.5%) women received a TA and 162,680 (67.5%) received an OI. In the TA group, 47,391 (60.6%) attended within six months of offered appointment and in the OI group 86,430 (53.1%) attended. This difference was significant (*p* < 0.001). The odds ratio (95% CI) for the attended outcome was 1.44 (1.33–1.55) adjusted for differences in deprivation and for invitation category (first invitation or subsequent invitation).

**Conclusions:**

This study supports the view that TA delivers a higher uptake than OI. It suggests that during this period over 12,000 women in London, who would have been expected to attend if given the standard TA, did not attend their appointment having received an OI.

## Introduction

The effect of the UK’s National Health Service Breast Screening Programme (NHSBSP) on breast cancer mortality depends in part upon good coverage, i.e., participation rates and timely screening. The NHSBSP invites all women aged 50–<71 for a routine screening once every 3 years. However, during the period April to June 2020 there was a hiatus in screening due to the Covid-19 pandemic and primary screening was limited. During the period July to September the screening services resumed, but at a reduced capacity, due to infection control restrictions. These two factors resulted in a backlog of invitations. In order to maximise the proportion of screening slots being used and reduce this backlog more quickly, a national decision was made to switch to issuing open invitations (OIs) rather than using the standard pre-booked timed appointment (TA) invitation. Prior to the pandemic, standard breast screening protocol was to offer eligible women a pre-booked TA with ∼2–3 weeks’ notice. In London, approximately 50–60% of women would be expected to attend these appointments and non-attenders were subsequently sent a second invite offering an alternative TA. Any women failing to respond to this were sent an OI, inviting them to contact the screening service to rebook. In contrast, the OI protocol simply invites the recipient to contact the screening service to book their first appointment and if there is no response after a specified time a second OI is sent out. OIs have been shown historically to result in lower breast screening uptake in the context of both first and second invitation letters^1–3^ but they lead to fewer unused appointment slots because a high proportion of women who respond to the OI by booking an appointment actually attend their chosen appointment.

Here we report on a study comparing uptake of the two types of invitation during a period in London when both types were in use at different screening sites. The findings of this study will provide the first large-scale, population-based data on the impact of different invitation types used during the Covid-19 pandemic recovery period.

## Methods

### Basic design

This study was designed as a natural experiment that retrospectively recorded the screening uptake of a large, ethnically and socially diverse population of over 200,000 women living in the London region, who received one of the two different routine breast screening invitation types. The aim was to determine whether there was a difference in uptake between the two invitation groups. The introduction of OIs in the London region began in the week commencing 30/10/2020. The change to OIs occurred screening site by screening site and the final batches of TA letters were sent out in early January 2021. The period September 2020 to March 2021 was therefore a transition period during which both appointment types were in circulation. This created conditions where both invitation types were in use at a similar time for similar populations. In anticipation of a reduction in uptake due to OIs, a number of interventions were deployed for recipients of OIs to try to increase uptake. Seven days after the initial invitation letter a text reminder was sent to each woman. At 14 days post-invite, a follow-up phone call was made to OI recipients who had not yet responded offering to book them an appointment. At four weeks post invite, the woman’s episode was closed if there had been still no response, and at this point a further OI letter was sent. All women with a booked appointment received a reminder of the date and time 48 h before the appointment. For recipients of TAs in the same period the same protocol was followed except that the “14-day” calls were omitted.

### Study participants

The study participants were all registered to GPs of the six ‘London’ breast screening services. The services are not individually identified in this study but given an identifier 1–6. The London Breast Screening Administration Hub (“the Hub”) hosted by The Royal Free London NHS Foundation Trust is responsible for processing around 400,000 routine invitations to women aged 50–<71 each year (including a call centre service). This means that the invitation protocols for all the study participants were subject to a large degree of standardisation. All six services using the Hub migrated to OI along the same time trajectory, shown in Supplementary Table 1. A single protocol was implemented for booking OIs, whereby these women were booked initially into a specified ‘dummy’ clinic. This enabled us to identify which women were in receipt of OIs and which received TAs. There was no reason to believe that a systematic difference existed between the characteristics of women in each group but to control for this, demographic data for each woman invited (age at appointment, ethnic group (where available) and Lower Super Output Area (LSOA) of residence) were collected from the six London National Breast Screening System databases that are used to manage the invitation process. The LSOA code was used to link other potentially relevant socio-economic data associated with the LSOA of residence, namely: the Index of Multiple Deprivation (IMD, 2019),^4^ % Black, Asian and Minority Ethnic (%BAME) and % Born outside of the UK (Office of National Statistics Census 2011).^5^

The date of first offered appointment (DOFOA) was defined as the booked appointment offered in the invitation letter for a TA. For an OI the DOFOA was the dummy clinic date (∼2–3 weeks after the OI was sent) or the actual appointment date where a woman responded to the OI. In addition, the category of the invitation was identified as follows: “First call” were women being invited for their first ever breast screening in the NHSBSP; “Persistent non-attenders” were women invited previously but who have never attended a previous appointment; “Recalls” were invites to previous attenders. It has been previously shown that uptake in Recall women is consistently higher than in the other groups.^6,7^

All women who had been selected for screening on or before 31/03/2021 and with a DOFOA on or after 01/09/2020 were included in the study. This range was designed in order that all women invited during the hybrid stage could be included. Self-referrals, early recalls and women under surveillance for a higher risk of breast cancer were excluded from the study.

Every woman has six months to respond to her invitation and therefore appointment and attendance data were downloaded from the six London National Breast Screening System databases in December 2021 when more than six months had elapsed after the final invitation date, thereby giving every woman time to respond. In all, 248,776 women aged between 50 and 71 were sent routine invitations during the study period. Of these, 7558 women were excluded from this analysis because they required ‘special’ appointments due to, for example, mobility issues. Special appointments are booked through a non-standard protocol that involves liaison with the client. The other reason for exclusion was because of an open episode (i.e., the outcome was not yet finalized; 530 women). This left a total of 240,872 women who were eligible for inclusion in the analysis (see Supplementary Table 2 for characteristics of the women excluded).

The single outcome of interest in this study was attendance at a screening appointment within 6 months of the date the episode was opened, which is the time frame used to calculate screening uptake. We did not differentiate between reasons for not attending screening.

### Ethical considerations

This retrospective study was carried out on fully anonymous, routinely collected data only, held in accordance with the NHS Cancer Screening Programmes Confidentiality and Disclosure Policy 2011. The NHSBSP has section 251 support under the NHS Act 2006. The study was conducted as part of routine audit data collection.

### Statistical methods

Logistic regression models were used to examine the strength of the association between the invitation method (OI or TA) and the odds of attending screening within 6 months of date the screening episode was opened. Robust standard errors (clustering by screening service) were used to account for the possibility that there were some similarities in the ways that women within a given service were treated, e.g., the 14-day telephone calls were carried out using local screening resources rather than the Hub.

An unadjusted model was run, and then two further adjusted models were run to control for factors that may influence screening uptake and that may differ slightly between the TA and the OI group. Firstly, we ran a minimally adjusted model to control for IMD and for the invitation category (Invite to previous non-attender, First ever Invite or Recall invite to previous attender). Secondly, a further adjusted model was run additionally adjusting for %BAME.

It has previously been shown that sociodemographic factors may influence response to screening invitation and therefore we tested whether the effect of the invitation methodology was modified by the IMD, %BAME and % Born Outside UK. The likelihood ratio test was used to assess significance of the terms in each model.

In all the analyses, we considered statistical significance (two-sided) at *p*-value < 0.05. All analyses were conducted in Stata (IC 14).

## Results

The characteristics of the 240,872 study subjects are shown in Table 1, which compares the characteristics of women in the two study groups. The mean age for the two groups was very similar (58.6 years and 58.4 years for TA and OI respectively). Among the 63% of the participants who reported their ethnicity (either at this attendance or a previous attendance), there were minor differences between proportions in each ethnic group; ∼48% were White in the TA group and ∼43% in the OI group. As noted before, these data were only available for screening attenders. Because of the poor availability of data for non-attenders, alternative estimates of ethnicity were adopted based on the characteristics of the LSOA where a woman lived. The mean LSOA-level %BAME in the TA group was 37.6% and in the OI group 36.3%, and the means for % born outside UK were 33.2% and 34.1% respectively. Likewise, there were small differences in the invitation type: 68% of the TA women were of type recall, whereas of the OIs, 67% were recalls. These between-group differences were relatively small but given their known a priori association with screening uptake these factors were controlled for in the fully adjusted logistic regression models for comparison. The balance of TA and OI was somewhat different across different screening services, but this was taken into account by using robust standard errors.

**Table 1.** Characteristics of study subjects.

	Received TA^a^	Received OI^a^
n = 240,872	n = 78,192 (32.5%)	n = 162,680 (67.5%)
**Age at first offered appointment**	**Mean (SD)**	**Mean (SD)**
	58.60 (5.87)	58.37 (5.90)
**Ethnicity** ^**b**^	**n (%)**	**n (%)**
White – British/Irish/other	34,190 (43.7%)	69,594 (42.8%)
Asian^c^	3707 (4.7%)	7712 (4.7%)
Black – British/Caribbean/other	8163 (10.4%)	13,642 (8.4%)
Black – African	973 (1.2%)	2225 (1.4%)
Mixed	791 (1.0%)	1872 (1.2%)
Chinese	3625 (4.6%)	7348 (4.5%)
Missing or not reported	26,743 (34.2%)	60,287 (37.1%)
**Average population classified as: Black, Asian & minority ethnic (BAME)** ^**d**^	**Mean (SD)**	**Mean (SD)**
	37.58% (21.04)	36.31% (18.95)
**Average population classified as: Born outside UK** ^**d**^	**Mean (SD)**	**Mean (SD)**
	33.16% (15.59)	34.13% (14.48)
**Invitation category** ^**e**^	**n (%)**	**n (%)**
Invite to persistent non-attender	13,939 (17.8%)	30,156 (18.5%)
First call	11,174 (14.3%)	23,676 (14.6%)
Recall	53,079 (67.9%)	108,848 (66.9%)
**Screening service**	**n (%)**	**n (%)**
1	22,167 (24.2%)	23,795 (25.7%)
2	10,948 (11.9%)	15,800 (17.0%)
3	12,869 (14.0%)	9564 (10.3%)
4	14,628 (16.0%)	9039 (9.7%)
5	18,549 (20.2%)	19,114 (20.6%)
6	12,500 (13.6%)	15,442 (16.6%)
**IMD quintile** ^f^	**n (%)**	**n (%)**
1	13,389 (14.6%)	13,629 (14.7%)
2	25,615 (27.9%)	22,576 (24.3%)
3	21,667 (23.6%)	21,557 (23.2%)
4	16,313 (17.8%)	19,486 (21.0%)
5	12,934 (14.1%)	13,238 (14.3%)
Not known	1743 (1.9%)	2268 (2.4%)

^a^ Women invited via standard invitation letter, either open invitation (OI) or timed appointment (TA). All non-attenders receive a further OI letter inviting them to call the office and book an appointment if they change their mind.

^b^ Ethnicity is only generally available for those who have attended at least one appointment.

^c^ Asian includes: British Indian, Pakistani, Bangladeshi and other.

^d^ Based on the LSOA data for the woman’s place of residence. The classification is provided by the ONS 2011 census data.^4^

^e^ Routine invitations only, i.e., high risk and self-referrals were excluded. First call are women invited for the first time. Persistent non-attenders are women invited previously but who have never attended a previous appointment. Recalls are invites to previous attenders.

^f^ Index of multiple deprivation (IMD) quintile based on woman’s home address (IMD 2019).^4^ NB: 1 is most deprived quintile.

 Table 2 shows numbers of women attending within each appointment group and the equivalent percentage uptake. Whilst raw uptake was 55.6% overall it was only 53.1% in the OI group and 60.6% in the TA group, a 7.5% difference in absolute terms (*P* < 0.001). Women receiving a TA were significantly more likely to attend compared to those who received an OI. The odds ratio (OR) of attending screening within 6 months in the TA group was 1.36 (095% CI 1.15–1.61) compared with the OI group without adjustment, and 1.44 (95% CI 1.34–1.54) adjusted for IMD, invitation type and %BAME. The confidence interval is considerably narrower after adjustment as the covariates account for much of the variation among clusters. The effect of the invitation type (TA or OI) did not vary by %BAME, % born outside UK or IMD quintile (results available from the authors).

**Table 2.** Attendance at screening unadjusted percentage uptake.

	Received TA^a^	Received OI^a^	All invitations
% Attended	n = 78,192	n = 162,680	n = 240,872
Yes	47,391 (60.61%)	86,430 (53.13%)	133,821 (55.6%)
No	30,801 (39.39%)	76,250 (46.87%)	107,051 (44.4%)
Absolute difference in attendance		−7.48%	

Pearson’s Χ^2^(1) = 1200 *P* < 0.001.

Notes: ^a^Women invited via standard invitation letter, either open invitation (OI) or timed appointment (TA). All non-attenders receive a further OI letter inviting them to call the office and book an appointment if they change their mind. These episodes are all more than 6 months old giving women maximum window of opportunity to attend, so are finalised uptake.

## Discussion

This study shows that in our study population of almost 250,000 women, those receiving a TA were significantly more likely to attend compared to those who received an OI, despite specific interventions to encourage the OI women to attend. Local area characteristics, deprivation, %BAME and % born outside UK did not significantly modify this effect.

This study lends weight to conclusions from other studies that a pre-booked TA is a more effective strategy for encouraging uptake of breast screening than offering OIs even, as in this case, when extra interventions have been added to support the OI recipients. If the TA invitation method had been used universally for the whole of the study group, it is estimated that a further ∼12,000 women would have attended during this period in whom ∼100 cancers could have been expected to be detected.

While these results arise from an observational, natural experiment, they are consistent with randomised trial results comparing TA with OI. This was observed in the context of invitations to first appointment^3^ and of repeat invitations to non-attenders.^2^ Thus, our results show that previous research setting findings are replicated in the setting of routine service screening.

Strengths of this study include its very large sample size relative to previous studies, and the socioeconomic and ethnic diversity of the population. Disadvantages include the fact that the design is essentially observational and therefore the study subjects were not randomized to the two invitation groups. As noted above, however, the results are consistent with previous randomized trial findings. Also, adjusting for covariates which might reasonably be expected to represent any bias between the two invitation groups did not annul or attenuate the effect. During the study period, England and London were subject to differences in the level of Covid lockdown at different times.^8^ Any possible differential effect of these lockdowns on the two invitation groups was not considered in this study since all women had the opportunity to delay their appointment irrespective of their invitation group. This study took place in London, which has traditionally had lower uptake rates than the rest of England. Thus, the absolute uptake rates cannot be generalized to the rest of the programme, but the relative increase in uptake with TA, as measured by the ORs above, is likely to be generalizable.

In conclusion, in an urban population of high ethnic and socioeconomic diversity, TAs were associated with a substantial and significant increase in uptake compared to OI.

## Supplemental Material

sj-docx-1-msc-10.1177_09691413221127583 - Supplemental material for Did the use of open invitations in place of timed appointment invitations reduce the uptake of breast screening in the London region during the COVID-19 recovery? Click here for additional data file. Supplemental material, sj-docx-1-msc-10.1177_09691413221127583 for Did the use of open invitations in place of timed appointment invitations reduce the uptake of breast screening in the London region during the COVID-19 recovery? by Sue M Hudson, Kathie Binysh and Stephen W Duffy in Journal of Medical Screening
