# Supplementary material for: Did the use of open invitations in place of timed appointment invitations reduce the uptake of breast screening in the London region during the COVID-19 recovery?
Source: J Med Screen. 2022 Oct 11;30(2):87–91. doi: 10.1177/09691413221127583 (PMC9554567; doi:10.1177/09691413221127583)
Supplement: sj-docx-1-msc-10.1177_09691413221127583 - Supplemental material for Did the use of open invitations in place of timed appointment invitations reduce the uptake of breast screening in the London region during the COVID-19 recovery? [file sj-docx-1-msc-10.1177_09691413221127583.docx]

**Supplementary Table 1. Timetable for switch from OI to TA across all London screening services**

| **Week commencing** | **Action** |
| --- | --- |
| 30th Oct 2020 | Commence using OIs for one screening site per screening service |
| 23rd November 2020 | Commence using OIs for a second screening site per service |
| 7th December 2020 | Commence using OIs for a third screening site per service |
| 4th January 2021 | Commence using OIs for all remaining sites per service |
| 31st March 2021 | 3 months since final batches of TA invitations were selected, giving reasonable time window for response |

**Supplementary Table 2. Characteristics of women excluded from study**

|  | Received TA ^a^ | Received OA ^a^ |
| --- | --- | --- |
| **n = 7904** | **n = 2491 (31.5%)** | **n = 5413 (68.5%)** |
|  |  |  |
| **Age at first offered appointment** | **Mean (SD)** | **Mean (SD)** |
|  | 59.46 (5.96) | 59.42 (5.93) |
|  |  |  |
| **Ethnicity** ^b^ | **n (%)** | **n (%)** |
| White – British/Irish/other | 1374 (55.2%) | 3077 (56.8%) |
| Asian ^c^ | 197 (7.9%) | 363 (6.7%) |
| Black – British/Caribbean/other | 187 (7.5%) | 363 (6.7%) |
| Black – African | 97 (3.9%) | 222 (4.1%) |
| Mixed | 50 (2.0%) | 87 (1.6%) |
| Chinese | 17 (0.7%) | 51 (0.9%) |
| Missing or not reported | 569 (22.8%) | 1250 (23.1%) |
|  |  |  |
|  |  |  |
| **Average population classified as: Black, Asian & minority ethnic (BAME)** ^d^ | **Mean (SD)** | **Mean (SD)** |
|  | 34.86% (19.65) | 34.67% (18.49) |
|  |  |  |
| **Average population classified as: Born outside UK** ^d^ | **Mean (SD)** | **Mean (SD)** |
|  | 32.04% (15.72) | 32.37% (14.28) |
|  |  |  |
| **Invitation category** ^e^  Invite to persistent non-attender  First call  Recall | **n (%)**  293 (11.8%)  219 (8.8%)  1979 (79.4%) | **n (%)**  522 (9.6%)  459 (8.5%)  4432 (81.9%) |
| **Screening service**  1  2  3  4  5  6 | **n (%)**  698 (28.0%)  283 (11.4%)  304 (12.2%)  300 (12.0%)  569 (22.8%)  337 (13.5%) | **n (%)**  1626 (30.0%)  519 (9.6%)  458 (8.5%)  539 (10.0%)  1276 (23.6%)  995 (18.4%) |
| **IMD quintile** ^f^  1  2  3  4  5  not known | **n (%)**  378 (15.2%)  728 (29.2%)  588 (23.6%)  480 (19.3%)  305 (12.2%)  12 (0.5%) | **n (%)**  1001 (18.5%)  1304 (24.1%)  1111 (20.5%)  1208 (22.3%)  755 (13.9%)  34 (0.6%) |

^a^  Women invited via standard invitation letter, either open invitation (OI) or timed appointment (TA). All non-attenders receive a further OI letter inviting them to call the office and book an appointment if they change their mind.

^b^  Ethnicity is only generally available for those who have attended at least one appointment.

^c^ Asian includes: British Indian, Pakistani, Bangladeshi and other.

^d^  Based on the LSOA data for the woman’s place of residence. The classification is provided by the ONS 2011 census data.^4^

^e^  Routine invitations only, i.e. high risk and self-referrals were excluded. First call are women invited for the first time. Persistent non-attenders are women invited previously but who have never attended a previous appointment. Recalls are invites to previous attenders.

^f^ Index of multiple deprivation (IMD) quintile based on woman’s home address (IMD 2019).^4^ NB: 1 is most deprived quintile.
